# Optimization of protoplast regeneration in the model plant *Arabidopsis thaliana*

**DOI:** 10.1186/s13007-021-00720-x

**Published:** 2021-02-23

**Authors:** Yeong Yeop Jeong, Hun-Young Lee, Suk Weon Kim, Yoo-Sun Noh, Pil Joon Seo

**Affiliations:** 1 Department of Chemistry, Seoul National University, Seoul, 08826 Korea; 2 Research Institute of Basic Sciences, Seoul National University, Seoul, 08826 Korea; 3 Department of Biological Sciences, Sungkyunkwan University, Suwon, 16419 Korea; 4 Plant Genomics and Breeding Institute, Seoul National University, Seoul, 08826 Korea; 5 Biological Resource Center, Korea Research Institute of Bioscience and Biotechnology, Jeongeup, 56212 Korea; 6 School of Biological Sciences, Seoul National University, Seoul, 08826 Korea

**Keywords:** Protoplast, Plant regeneration, Pluripotency, Cell division, De novo organogenesis

## Abstract

**Background:**

Plants have a remarkable reprogramming potential, which facilitates plant regeneration, especially from a single cell. Protoplasts have the ability to form a cell wall and undergo cell division, allowing whole plant regeneration. With the growing need for protoplast regeneration in genetic engineering and genome editing, fundamental studies that enhance our understanding of cell cycle re-entry, pluripotency acquisition, and de novo tissue regeneration are essential. To conduct these studies, a reproducible and efficient protoplast regeneration method using model plants is necessary.

**Results:**

Here, we optimized cell and tissue culture methods for improving protoplast regeneration efficiency in *Arabidopsis thaliana*. Protoplasts were isolated from whole seedlings of four different *Arabidopsis* ecotypes including Columbia (Col-0), Wassilewskija (Ws-2), Nossen (No-0), and HR (HR-10). Among these ecotypes, Ws-2 showed the highest potential for protoplast regeneration. A modified thin alginate layer was applied to the protoplast culture at an optimal density of 1 × 10^6^ protoplasts/mL. Following callus formation and de novo shoot regeneration, the regenerated inflorescence stems were used for de novo root organogenesis. The entire protoplast regeneration process was completed within 15 weeks. The in vitro regenerated plants were fertile and produced morphologically normal progenies.

**Conclusion:**

The cell and tissue culture system optimized in this study for protoplast regeneration is efficient and reproducible. This method of *Arabidopsis* protoplast regeneration can be used for fundamental studies on pluripotency establishment and de novo tissue regeneration.

## Background

Plants have a remarkable reprogramming potential, which facilitates plant regeneration from organs, tissues, and even a single cell. Protoplasts exhibit a remarkable ability to dedifferentiate, and cultured protoplasts have the ability to form cell walls and undergo cell division, allowing whole plant regeneration [1, 2]. Given the advantages of improved synchrony initiated from a single cell without sexual reproduction, protoplast regeneration techniques have been widely used for genetic engineering and genome editing in plants. For example, the clustered regularly interspaced short palindromic repeat (CRISPR)/CRISPR-associated protein 9 (Cas9) system has been transiently expressed in plant protoplasts, and genome-edited protoplasts have been regenerated into individual plants [3]. Moreover, DNA-free genome editing has been developed with the delivery of preassembled Cas9-gRNA ribonucleoproteins (RNPs) into protoplasts derived from somatic tissues [4]. The RNPs have been successfully introduced into various plant species, such as *Arabidopsis thaliana*, tobacco (*Nicotiana attenuata*), lettuce (*Lactuca sativa* L.), rice (*Oryza sativa* L.), petunia (*Petunia* × *atkinsiana*), and potato (*Solanum tuberosum* L.), via polyethylene glycol–calcium (PEG–Ca^2+^)-mediated transfection [5–9].

Fundamental studies on key processes involved in protoplast regeneration, including cell wall recovery, cell cycle re-entry, callus formation, pluripotency acquisition, and de novo tissue regeneration, are essential. Protoplast regeneration is distinct from tissue explant-derived plant regeneration [10, 11], and molecular processes involved in cell fate transition during protoplast regeneration are largely unknown. Understanding the molecular mechanisms underlying protoplast regeneration will further advance plant cell-based biotechnological applications, such as genome editing and somatic cell hybridization.

Protoplast regeneration methods have been developed in several plant species. Conventional methods of protoplast regeneration involve liquid culture. Liquid culture of protoplasts is a simple and easy technique used to induce cell division and callus formation, but it has a low efficiency of tissue regeneration, owing to cell aggregation-induced cell death and low cell proliferation activity [12–14]. To overcome the limitations of liquid culture, several studies have developed protoplast-embedding methods for protoplast immobilization using low-melting agarose and alginate [12]. Alginate-embedding methods have been widely used for protoplast regeneration. Alginate forms a hydrogel via crosslinking with divalent alkaline metal ions, such as strontium (Sr^2+^), barium (Ba^2+^), and calcium (Ca^2+^) [15]. Alginate hydrogels can immobilize protoplasts and maintain their viability for proliferation without aggregation. The Ca^2+^-alginate embedding method has been successfully applied for protoplast regeneration in various plant species, including tobacco, *Arabidopsis*, *Brassica*, petunia, lotus, and barley [13, 14, 16–20].

Although several protoplast regeneration methods have been reported previously [17, 21–24], the application of these methods is not reproducible, particularly in the model plant *Arabidopsis*. An improved protoplast regeneration protocol for *Arabidopsis*, with high reproducibility and efficiency, is necessary for genetic, biochemical, and molecular biology studies. Here, we screened four *Arabidopsis* ecotypes, among which Wassilewskija (Ws-2) showed the highest protoplast regeneration ability. In vitro culture conditions for initial cell division, microcallus formation, and de novo shoot and root regeneration were further optimized. The entire protoplast regeneration protocol could be reproducibly completed within 15 weeks. The improved method of protoplast regeneration developed in this study provides opportunities for fundamental studies on cell proliferation, cellular pluripotency, and de novo tissue regeneration.

## Methods

### Chemicals and equipment

All chemicals were purchased from Sigma-Aldrich, Duchefa Biochemie, Merck, Novozymes, and Junsei (Additional file 1). Equipments used in this study are listed in Additional file 2.

### Reagents

#### MMC (10 mM MES, 0.47 M Mannitol, 10 mM Calcium) solution

To prepare the MMC solution, 85 g d-mannitol, 2.132 g 2-morpholinoethanesulfonic acid monohydrate (MES·H_2_O), and 1.11 g CaCl_2_ were dissolved in 1 L double distilled water (ddH_2_O). The pH of the MMC solution was adjusted to 5.8 with 2 M NaOH and/or 1 M HCl. The solution was sterilized in an autoclave at 121 °C for 10 min and stored at room temperature.

#### Enzyme solution

To prepare the enzyme solution, 1 mL of Viscozyme L, 0.5 mL of Celluclast 1.5 L, and 0.5 mL of Pectinex ultra SP-L were added to 48 mL of the MMC solution. The solution was sterilized using a 0.2-µm syringe filter.

#### Sucrose solution (0.6 M)

To prepare 0.6 M sucrose, 205.38 g sucrose and 0.42 g MES·H_2_O were dissolved in 1 L ddH_2_O. The pH was adjusted to 5.8 with 2 M NaOH and/or 1 M HCl. The solution was sterilized by autoclaving at 121 °C for 10 min and then stored at room temperature.

#### Mannitol solution (0.5 M)

To prepare 0.5 M mannitol, 91 g d-mannitol and 0.42 g MES·H_2_O were dissolved in 1 L ddH_2_O. The pH was adjusted to 5.8 with 2 M NaOH and/or 1 M HCl. The solution was sterilized in an autoclave at 121 °C for 10 min and stored at room temperature.

#### Sodium alginate solution

To prepare sodium alginate solution, 2.8 g sodium alginate and 7.28 g d-mannitol were dissolved in 100 mL of ddH_2_O. The solution was first sterilized in an autoclave at 121 °C for 10 min and stored at room temperature. Any precipitates in the sodium alginate solution were further removed using a 0.2 µm syringe filter before use.

#### CaCl_2_-agar

To prepare CaCl_2_-agar, 72.8 g d-mannitol and 2.2 g CaCl_2_ were dissolved in 1 L ddH_2_O. The 10 g plant agar was added to the prepared solution. The final solution was autoclaved at 121 °C for 10 min and stored at 4 °C.

#### CaCl_2_ solution (50 mM)

To prepare CaCl_2_ solution, 72.8 g d-mannitol and 5.5 g CaCl_2_ were dissolved in 1 L ddH_2_O. The solution was sterilized in an autoclave at 121 °C for 10 min and stored at room temperature.

#### Cell and tissue culture media

All liquid media were sterilized using a 0.2 µm syringe filter. The composition of all solutions and culture media is summarized in Table 1.

**Table 1:** Composition of culture media used in this study for *Arabidopsis* protoplast regeneration

Medium name	Medium composition	Storage	Function	References
Protoplast Induction Medium (PIM)	Gamborg B5 medium containing vitamins, 20 g/L sucrose, 60 g/L myo-inositol, 2 mg/L 6-BAP, and 0.5 mg/L α-NAA (pH adjusted to 5.8 using 2 M NaOH or 1 M HCl). Medium was sterilized using a 0.2 µm syringe filter	Freshly prepared	Induction of protoplast division	
Culture Medium A (CMA)	Gamborg B5 medium containing vitamins, 72 g/L d-glucose, 1 mg/L 2,4-D, and 0.15 mg/L 6-BAP (pH adjusted to 5.8 using 2 M NaOH or 1 M HCl). Medium was sterilized using a 0.2 µm syringe filter	Freshly prepared	Induction of protoplast division	[17]
Protoplast Culture *Arabidopsis* (PCA)	Gamborg B5 medium containing vitamins, 85 g/L d-glucose, 0.75 g/L MgSO_4_^.^7H_2_O, 0.34 g/L CaCl_2_, 0.05 g/L l-glutamine, 20 mL/L coconut water, 0.1 mg/L 2-IP, and 0.5 mg/L α-NAA (pH adjusted to 5.8 using 2 M NaOH or 1 M HCl). Medium was sterilized using a 0.2 µm syringe filter	Freshly prepared	Induction of protoplast division; microcallus growth	[21]
Callus Induction Medium (CIM)	Gamborg B5 medium containing vitamins, 20 g/L sucrose, 2 mg/L 6-BAP, and 0.5 mg/L α-NAA (pH adjusted to 5.8 using 2 M NaOH or 1 M HCl). Medium was sterilized using a 0.2 µm syringe filter	Freshly prepared	Microcallus growth	
Culture Medium C (CMC)	Murashige and Skoog (MS) medium containing vitamins, 54 g/L d-glucose, 20 g/L sucrose, 0.5 g/L myo-inositol, 0.45 g/L l-glutamine, 0.05 mg/L 2,4-D, and 1 mg/L kinetin (pH adjusted to 5.8 using 2 M NaOH or 1 M HCl). Medium was sterilized using a 0.2 µm syringe filter	Freshly prepared	Microcallus growth	[17]
Shoot Induction Medium (SIM)	MS medium containing vitamin, 30 g/L sucrose, 0.47 g/L MES^.^H_2_O, 0.1576 mg/L IAA, and 0.501 mg/L 2-IP. The pH was adjusted to 5.8 using 2 M NaOH or 1 M HCl before the addition of plant agar (8 g/L), and the medium was sterilized by autoclaving at 121 °C for 10 min	Freshly prepared	Induction of shoot regeneration	[29]
Shoot Regeneration Medium A (SRMA)	MS medium containing vitamins, 20 g/L sucrose, 0.47 g/L MES^.^H_2_O, 7 mg/L 2-IP, and 0.05 mg/L IAA. The pH was adjusted to 5.8 using 2 M NaOH or 1 M HCl before the addition of plant agar (8 g/L), and the medium was sterilized by autoclaving at 121 °C for 10 min	Freshly prepared	Induction of shoot regeneration	[17]
Shoot Regeneration *Arabidopsis* (SRA)	Half-strength MS (1/2 MS) medium containing vitamin, 15 g/L sucrose, 0.47 g/L MES^.^H_2_O, 2 mg/L kinetin, and 0.05 mg/L IAA. The pH was adjusted to 5.8 using 2 M NaOH or 1 M HCl before the addition of plant agar (8 g/L), and the medium was sterilized by autoclaving at 121 °C for 10 min	Freshly prepared	Induction of shoot regeneration	[23]
Murashige and Skoog Medium (MS)	1/2 MS medium containing vitamin, 10 g/L sucrose, and 0.47 g/L MES^.^H_2_O. The pH was adjusted to 5.8 using 2 M NaOH or 1 M HCl before the addition of plant agar (8 g/L), and the medium was sterilized by autoclaving at 121 °C for 10 min	Freshly prepared	Induction of root emergence; seedling growth	[17]
Rooting Medium (RM)	1/2 MS medium containing vitamin, 10 g/L sucrose, 0.47 g/L MES^.^H_2_O, and 1 mg/L IBA. The pH was adjusted to 5.8 using 2 M NaOH or 1 M HCl before the addition of plant agar (8 g/L), and the medium was sterilized by autoclaving at 121 °C for 10 min	Freshly prepared	Induction of root emergence	[17]
Root Regeneration *Arabidopsis* (RRA)	MS medium containing vitamin, 15 g/L sucrose, 0.47 g/L MES^.^H_2_O, 1 mg/L α-NAA, and 0.5 mg/L IBA. The pH was adjusted to 5.8 using 2 M NaOH or 1 M HCl before the addition of plant agar (8 g/L), and the medium was sterilized by autoclaving at 121 °C for 10 min	Freshly prepared	Induction of root emergence	[21]

### Plant materials and growth conditions

Four ecotypes of *Arabidopsis thaliana*, including Columbia (Col-0), Wassilewskija (Ws-2), Nossen (No-0), and HR (HR-10), were used in this study unless specified otherwise. *Arabidopsis* seeds were sterilized and sown on a half-strength Murashige and Skoog (1/2 MS) medium supplemented with 1% sucrose and 0.8% agar. Plates were incubated at 22–23 °C under long-day (LD) photoperiod (16 h light/8 h dark) and 100 µmol photons m^−2^ s^−1^ light intensity using cool white fluorescent lamps.

### Protoplast isolation

Ten-day-old whole seedlings of all four *Arabidopsis* ecotypes were soaked in 20 mL of 0.5 M mannitol at 22–23 °C for 1 h. Then, 0.5 M mannitol was replaced with 20 mL enzyme solution, and the seedlings were incubated in the dark at room temperature for 12 h, with gentle shaking (50 rpm). Undigested tissues were removed by filtering the sample through 40 µm cell strainers. Protoplasts were collected by centrifugation at 100×*g* for 7 min at room temperature. The protoplast-containing pellet was resuspended in 2 mL MMC solution, and the protoplast suspension was overlaid on 6 mL of 0.6 M sucrose. The resulting sample was centrifuged at 80×*g* for 10 min at room temperature. Protoplasts at the sucrose–MMC interface were gently transferred to a new 14 mL round-bottom tube. The purified protoplasts were washed twice with 0.5 M mannitol by centrifugation at 100×*g* for 5 min at room temperature. After resuspension in 0.5 M mannitol solution, the protoplast cell number was counted under a light microscope using a hemocytometer. The final protoplast density was adjusted to 2 × 10^6^ protoplasts/mL.

### Ca^2+^-alginate embedding

Immobilization of *Arabidopsis* protoplasts in Ca^2+^-alginate was performed as described previously [17], with several modifications. Protoplast suspension was mixed with an equal volume of sodium alginate solution to obtain a final density of 1 × 10^6^ protoplasts/mL. Then, 2 mL protoplast–alginate mixture was gently spread onto CaCl_2_-agar in a 60 mm Petri dish (21.50 cm^2^ area). After 1 h incubation at room temperature, a layer of alginate hydrogel containing immobilized protoplasts was formed. Then, 2 mL CaCl_2_ solution was applied onto the alginate hydrogel and incubated for 30 min to complete polymerization. A quarter of the hydrogel (5.375 cm^2^ area) was transferred to a 60 mm Petri dish containing 4 mL liquid medium for protoplast proliferation [Protoplast Induction Medium (PIM), Culture Medium A (CMA), or Protoplast Culture Arabidopsis (PCA)]. Protoplasts were incubated in the dark at 25 °C.

### Microcolony regeneration

The protoplast–alginate hydrogel was incubated for 4 weeks in liquid media for protoplast proliferation [PIM, CMA, or PCA]. Then, the liquid medium was replaced by 4 mL liquid medium for callus proliferation [Callus Induction Medium (CIM), Culture Medium C (CMC), or Protoplast Culture *Arabidopsis* (PCA)]. The plates were incubated for 3 weeks under continuous dim light conditions (15 µmol photons m^−2^ s^−1^) at 25 °C, allowing the formation of microcalli. Microcalli (diameter > 1 mm) were picked and transferred to solid media for de novo shoot regeneration [Shoot Induction Medium (SIM), Shoot Regeneration Medium A (SRMA), or Shoot Regeneration *Arabidopsis* (SRA)]. After incubation at 25 °C under continuous light conditions (50 µmol photons m^−2^ s^−1^) for 3 weeks, the regenerated shoots were excised and transferred to solid media for de novo root regeneration [MS, Rooting Medium (RM), or Root Regeneration *Arabidopsis* (RRA)]. After 3 weeks, the rooted plantlets were transferred to soil and grown further at 23 °C under LD photoperiod and 100 µmol photons m^−2^ s^−1^ light intensity (cool white fluorescent lamps).

## Results

### Cell wall digestion of whole Arabidopsis seedlings

**Fig. 1:**
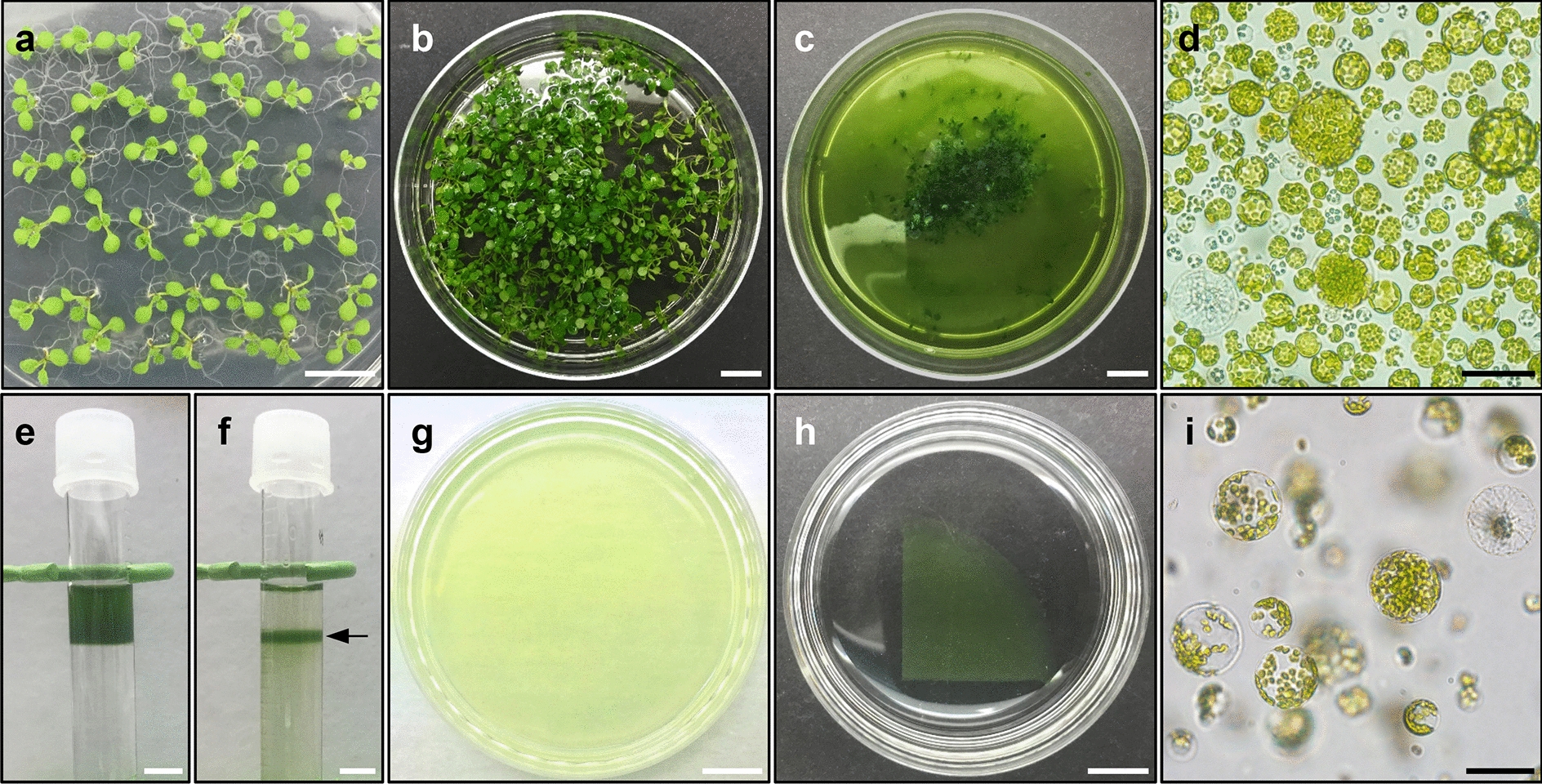
Protoplast isolation from *Arabidopsis* seedlings. **a** Ten-day-old seedlings grown under long-day (LD; 16 h light/8 h dark) conditions. **b** Preplasmolysis of seedlings in 0.5 M mannitol. **c** Protoplast isolation in enzyme solution. **d** Microscope image of isolated protoplasts at 12 h post-incubation in enzyme solution. **e** Overlaying protoplast solution onto 0.6 M sucrose during sucrose density gradient-based protoplast purification. **f** Viable protoplasts in the middle of the sucrose gradient solution after centrifugation. Arrow indicates the protoplast layer. **g** Immobilization of protoplasts in Ca^2+^-alginate hydrogel. **h** Protoplast culture in 4 mL PIM. **i** Protoplasts in PIM-incubated alginate hydrogel. White scale bars = 1 cm; black scale bars = 50 µm

Protoplast isolation is an important first step in the protoplast regeneration protocol that determines protoplast yield and quality and also influences the subsequent steps. Here, we optimized the *Arabidopsis* protoplast isolation protocol based on previous reports [17]: (1) we employed four *Arabidopsis* ecotypes (Col-0, Ws-2, No-0, and HR-10), which exhibit different tissue explant-derived plant regeneration capabilities (Additional file 3) [25, 26], and identified the ecotype that shows the best performance; (2) keeping in mind user-convenience, our protocol was optimized for protoplast isolation from 10-day-old whole seedlings (Fig. 1a); (3) plasmolysis was conducted in 0.5 M mannitol for 1 h to ensure high protoplast yield [17] (Fig. 1b); and (4) the preplasmolysed seedlings were incubated in enzyme solution for 12 h with gentle shaking [9] (Fig. 1c).

Optimal incubation time in enzyme solution is important. Based on our protocol, 400–500 seedlings (1–1.2 g fresh weight) yielded 1–2 × 10^7^ protoplasts (Fig. 1d), regardless of the *Arabidopsis* ecotype, after incubation in 20 mL enzyme solution for 12 h. Longer incubation (> 16 h) resulted in fragile protoplasts with low division potentials.

#### Cell wall digestion protocol

All of the following steps should be conducted in a sterile condition, and all solutions and materials must be sterilized to avoid contamination.

1. Soak *Arabidopsis* seeds in 1 mL of 75% ethanol solution containing 0.03% Triton X-100 for 10 min. Then, rinse the seeds with 1 mL of 70% ethanol twice for 5 min each time. After drying, sow the sterilized seeds on MS medium (100 mm Petri dish) and cold-stratify for 3 days at 4 °C.
  Note: Ethanol wash is sufficient for the sterilization of *Arabidopsis* seeds.
2. Germinate and grow seedlings at 23 °C under LD photoperiod and 100 µmol photons m^−2^ s^−1^ light intensity with cool white fluorescent lamps as the light source.
3. To perform pre-plasmolysis, transfer 400–500, 10-day-old seedlings to a 90 mm Petri dish containing 20 mL of 0.5 M mannitol using forceps, and seal the Petri dish with parafilm.
  Note: Seedlings should be perfectly submerged in 0.5 M mannitol for efficient protoplast isolation.
4. Incubate the Petri dish at room temperature (RT) for 1 h without shaking.
5. Replace the 0.5 M mannitol with 20 mL enzyme solution.
6. Incubate the Petri dish at RT in darkness for at least 10 h, with gentle shaking at 50 rpm.
  Note 1: Prolonged incubation in enzyme solution results in fragile protoplasts. When incubation time exceeds 16 h, protoplast solution turns brown. Therefore, incubation for 12 h is recommended.
  Note 2: 400–500 seedlings yield approximately 1–2 × 10^7^ protoplasts.

### Protoplast isolation and embedding in Ca^2+^-alginate hydrogels

The cell wall digestion allowed to collect protoplasts originated mainly from cotyledons and rosette leaves. While the isolated *Arabidopsis* protoplasts were heterogeneous, a small portion of protoplasts likely had competence to induce microcallus formation; however, these protoplasts usually have a low cell proliferation activity [10, 27], which is a major hurdle for protoplast regeneration. To overcome this limitation, we enriched the viable protoplasts using sucrose density gradient purification methods [22] (Fig. 1e, f).

Isolated protoplasts should be subjected to optimal culture conditions. Several cell culture methods have been suggested to induce the division of isolated protoplasts [12]. However, liquid culture methods showed limitations in our conditions, which resulted in higher frequencies of cell aggregation and cell death. In contrast, protoplast embedding in hydrogels led to higher cell survival and proliferation. In particular, alginate-based protoplast embedding showed the best performance for protoplast cultures [17, 24]. We therefore optimized a protoplast embedding method using Ca^2+^-alginate mixture. A main technical concern was the irregular thickness of Ca^2+^-alginate hydrogel, which affects the cell proliferation rate, owing to irregular cell density and cell respiration [12, 28]. To synthesize a reliable Ca^2+^-alginate hydrogel with regular thickness and the capacity to produce reproducible results, we improved a conventional protocol. Two milliliters of protoplast–alginate mixture was poured onto a 60 mm CaCl_2_-agar plate, resulting in hydrogels with a uniform diameter (60 mm) and thickness (0.5 mm) (Fig. 1g–i).

In addition, consistent with the previous finding that protoplast density in the hydrogel was an important determinant for initial cell division, we found that approximately 1 × 10^6^ protoplasts/mL was an optimal protoplast density in the protoplast culture (Additional file 4), but lower or higher cell density interfered with protoplast proliferation [17, 24].

#### Protoplast isolation and embedding protocol

1. Filter the protoplasts immersed in 20 mL enzyme solution using 40 µm cell strainers to remove undigested tissues and debris, and collect the filtrate in a 90 mm Petri dish.
  Note: Examine the filtered protoplasts under a light microscope to confirm their yield and intactness.
2. Split the filtrate equally into two 14 mL round-bottom tubes, and adjust final volume to 12 mL in each tube using the MMC solution.
3. Centrifuge the tubes using a swing-bucket rotor at 100×*g* for 7 min at RT.
4. Remove the supernatant.
  Note: The supernatant does not need to be completely removed. Residual volume < 100 µL is acceptable.
5. Carefully resuspend the pellet in each tube with 2 mL MMC solution.
6. Add 6 mL of 0.6 M sucrose in two new 14 mL round-bottom tubes each.
7. Carefully overlay 2 mL protoplast suspension onto 0.6 M sucrose.
8. Centrifuge the samples using swing-bucket rotor at 80×*g* for 10 min at RT.
9. Transfer 2 mL purified protoplasts from each tube into two new 14 mL round-bottom tubes; intact protoplasts will be suspended at the sucrose–MMC interface.
10. Adjust the volume to 10 mL with 0.5 M mannitol in each tube, and resuspend the protoplasts.
11. Centrifuge the samples using a swing-bucket rotor at 100×*g* for 5 min at RT.
12. Remove the supernatant.
13. Resuspend protoplasts in 10 mL of 0.5 M mannitol in each tube, and count the number of protoplasts in each tube under a microscope using a hemocytometer.
14. Centrifuge the samples using a swing-bucket rotor at 100×*g* for 5 min at RT.
15. Remove the supernatant.
  Note: The supernatant should be removed completely. Residual Ca^2+^ ions present in the MMC solution will result in premature polymerization of the protoplast–alginate mixture.
16. Resuspend the protoplasts in 0.5 M mannitol to a concentration of 2 × 10^6^ protoplasts/mL.
17. Mix 1 mL protoplast suspension (2 × 10^6^ protoplasts) gently with 1 mL of 2.8% sodium alginate solution for alginate hydrogel formation.
  Note: The final protoplast density in the protoplast–alginate mixture should be in the range of 0.5–1 × 10^6^ protoplasts/mL; do not exceed 1 × 10^7^ protoplasts/mL.
18. Pour the 2 mL protoplast–alginate mixture onto CaCl_2_-agar in a 60 mm Petri dish, and incubate the plate at RT for 1 h.
  Note: The alginate layer should be uniformly thin (~ 0.5 mm) throughout the gel.
19. Pour another 2 mL aliquot of CaCl_2_ solution onto the alginate hydrogel, and incubate the plate for 30 min at RT to perfectly solidify the gel.

### Protoplast division and microcallus formation

**Fig. 2:**
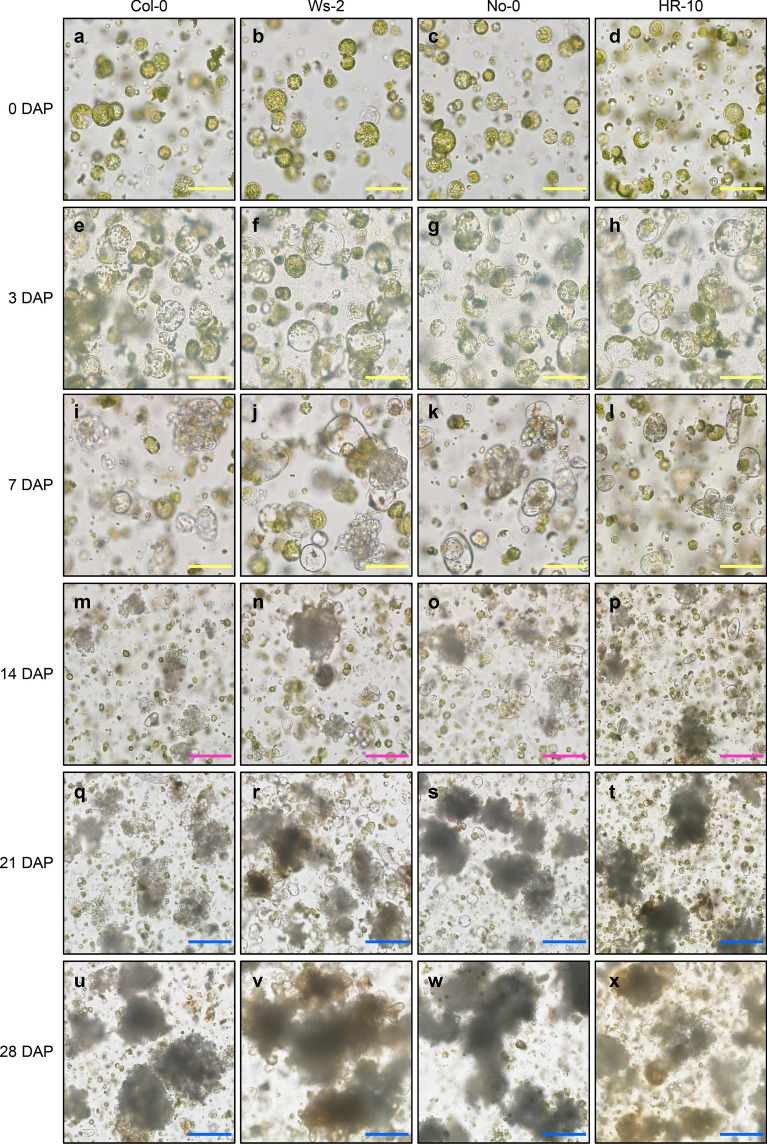
Division of *Arabidopsis* protoplasts in PIM. **a**–**x** Protoplast images taken at the indicated time points (days) after incubation in PIM (DAP): 0 DAP (**a**–**d**), 3 DAP (**e**–**h**), 7 DAP (**i**–**l**), 14 DAP (**m**–**p**), 21 DAP (**q**–**t**), and 28 DAP (**u**–**x**). Yellow scale bars = 100 µm; magenta scale bars = 200 µm; blue scale bars = 250 µm

A small fraction of protoplasts proliferated, although the relevant cell types are currently elusive. The re-entry of the protoplasts into the cell cycle is followed by colony and microcallus formation. Protoplast swelling was observed at 3–4 days after incubation on PIM (DAP) (Fig. 2a–h), and the first cell division was observed at approximately 7 DAP (Fig. 2i–l). A proliferating protoplast colony (diameter =  ~ 200 µm) was observed at approximately 14 DAP (Fig. 2m–p). The protoplast colony was grown into microcalli (Fig. 2q–t), which reached a size of 0.6 mm in diameter at ~ 28 DAP (Fig. 2u–x), while most protoplasts remained in a non-proliferative state.

To identify a culture medium optimal for the proliferation of *Arabidopsis* protoplasts, we tested protoplast proliferation rates in three different liquid media, including PIM, CMA [17], and PCA [21] (Table 2). No significant differences were detected in the protoplast proliferation rate among the four *Arabidopsis* ecotypes in each medium, except for HR-10 in CMA (Table 2). However, all ecotypes displayed higher protoplast proliferation rate in PIM, and relatively low proliferation in CMA and PCA media at 2 weeks after incubation (Table 2). These results indicate that PIM is optimal for protoplast proliferation.

**Table 2:** Percentage of dividing protoplasts of four different *Arabidopsis* ecotypes in three different media

Ecotypes	Media		
	PIM (protoplasts mm^−2^)	CMA (protoplasts mm^−2^)	PCA (protoplasts mm^−2^)
Col-0	14.83 ± 3.84	14.71 ± 2.06	2.53 ± 0.96
Ws-2	16.32 ± 2.21	13.10 ± 3.48	2.18 ± 0.85
No-0	13.68 ± 3.34	10.92 ± 2.63	1.49 ± 0.87
HR-10	15.75 ± 3.56	1.84 ± 1.49	5.86 ± 1.49

Protoplasts were embedded in Ca^2+^-alginate hydrogels, which were incubated in three different media for 2 weeks. The number of dividing protoplasts in a unit area of hydrogel was calculated. Data represent the mean ± SEM of biological triplicates

To induce de novo shoot formation at the later step, the callus size should be at least 0.8 mm in diameter (microcallus formation) [17]. While PIM was optimal for cell division at the initial stages of protoplast proliferation, it was sub-optimal for the formation of microcallus. Thus, after a 4-week-incubation, PIM was replaced by liquid medium for callus proliferation. To optimize the protocol, we prepared three different media, including CIM, CMC [17], and PCA [21], and determined the efficiency of callus formation (Table 3). As a result, a 2-week-incubation in CIM resulted in higher callus formation efficiency compared with that in CMC and PCA (Table 3).

**Table 3:** Efficiency of callus formation from Ca^2+^-alginate hydrogel-embedded protoplasts

Ecotypes	Media		
	CIM (Calli cm^−2^)	CMC (Calli cm^−2^)	PCA (Calli cm^−2^)
Col-0	5.75 ± 0.62	4.96 ± 0.35	6.34 ± 0.44
Ws-2	6.63 ± 0.40	4.88 ± 0.29	3.09 ± 0.57
No-0	6.11 ± 0.43	4.45 ± 0.46	6.43 ± 0.35
HR-10	6.37 ± 0.16	5.42 ± 0.30	4.04 ± 0.10

The hydrogels were incubated in PIM, followed by a 2-week incubation in three different media for callus proliferation. The number of dividing calli with a diameter of > 1 mm in a fixed area of hydrogel area (21.50 cm^2^) was measured. Data represent the mean ± SEM of biological triplicates

**Fig. 3:**
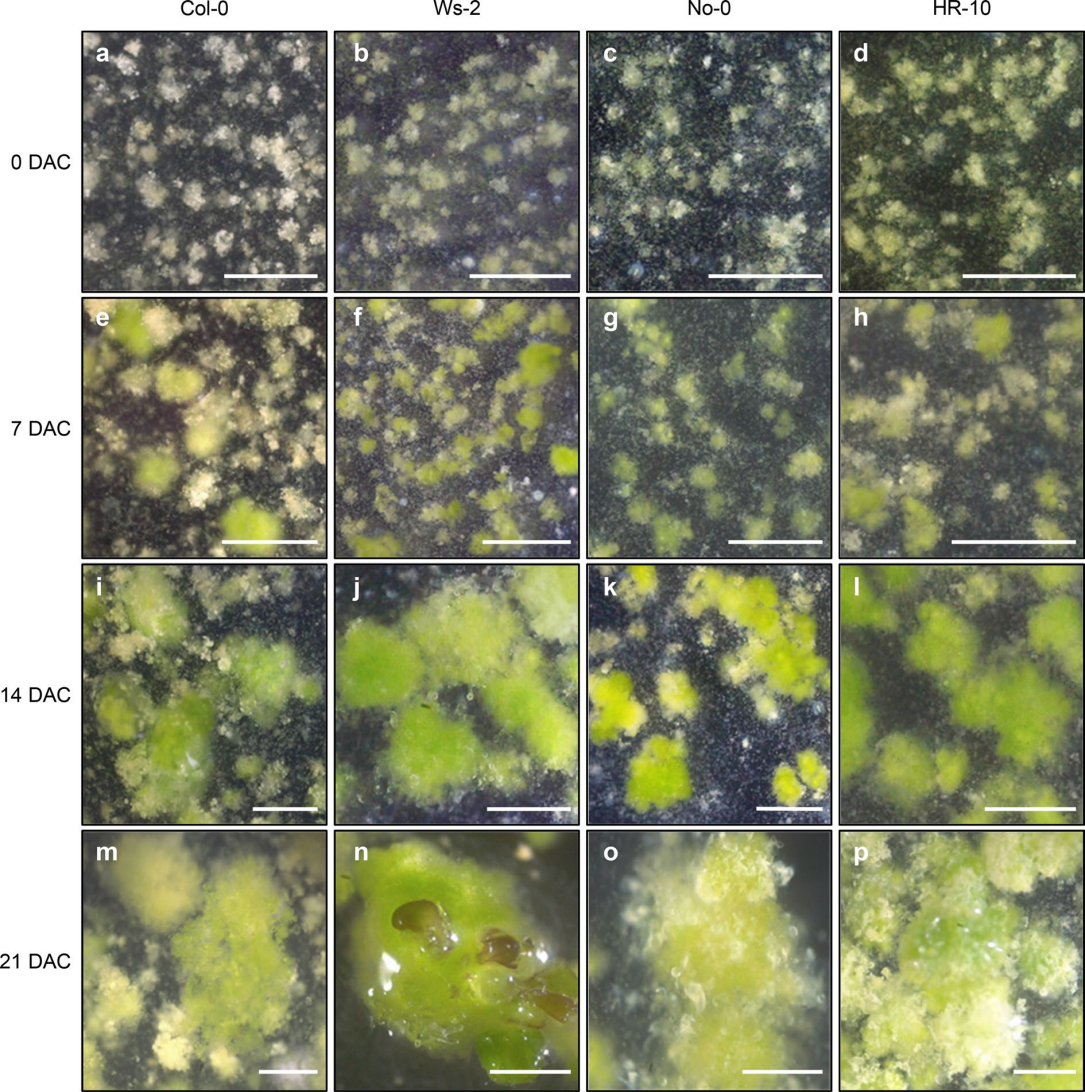
Microcalli formation in CIM. **a**–**p** Images of callus taken at the indicated time points (days) after incubation in CIM (DAC): 0 DAC (**a**–**d**), 7 DAC (**e**–**h**), 14 DAC (**i**–**l**), and 21 DAC (**m**–**p**). Scale bars = 1 mm

Microcalli reached a diameter of 1–2 mm after incubation in liquid CIM for 2 weeks (Fig. 3a–p). Notably, *Arabidopsis* ecotypes displayed differential callus formation capabilities (Table 3). In CIM, Ws-2 protoplasts efficiently produced calli, which further showed de novo shoot regeneration. On average, about 44.5% of callus with a size above 1 mm in a diameter produced regenerated leaves at 3 weeks after incubation in CIM (Table 4). Shoot regeneration in CIM was possible because of pre-incubation in cytokinin-rich PIM. Furthermore, HR-10 displayed a similar level of callus formation but lower efficiency of shoot regeneration in CIM, whereas No-0 and Col-0 had no shoot regeneration potential, compared with Ws-2 (Table 4). Taken together, we optimized the cell and tissue culture media and identified the *Arabidopsis* ecotype with the highest callus formation efficiency.

**Table 4:** Shoot regeneration efficiency in media for callus formation

Ecotypes	Media		
	CIM (%)	CMC (%)	PCA (%)
Col-0	N.D	N.D	N.D
Ws-2	44.50 ± 2.69	4.91 ± 1.66	N.D
No-0	N.D	N.D	N.D
HR-10	0.51 ± 0.16	N.D	N.D

Shoot regeneration rate was calculated as the percentage of calli with shoots relative to the number of microcalli with a size of > 1 mm in a diameter. The percentage was measured at 3 weeks after incubation in each medium. Data represent mean ± SEM of biological triplicates

*N.D* not determined

#### Microcallus formation protocol

1. Cut the protoplast–alginate gel using a sterilized scalpel blade, and transfer a quarter of the gel to a 60 mm Petri dish containing 4 mL liquid PIM using a sterilized spatula (Fig. 1h, i).
2. Seal the plates with parafilm and incubate the protoplast–alginate gel at 25 °C in darkness for 4 weeks. Add fresh PIM to a final volume of 4 mL every 2 weeks.
  Note: After a 3-week incubation in PIM, proliferating protoplast colonies reach a size of 300–500 μm in diameter.
3. Replace PIM with 4 mL CIM.
4. Incubate the alginate gel for 3 weeks at 25 °C under continuous dim light conditions (15 µmol photons m^−2^ s^−1^). Add fresh CIM to a final volume of 4 mL every 2 weeks.

### Shoot regeneration from microcallus

**Fig. 4:**
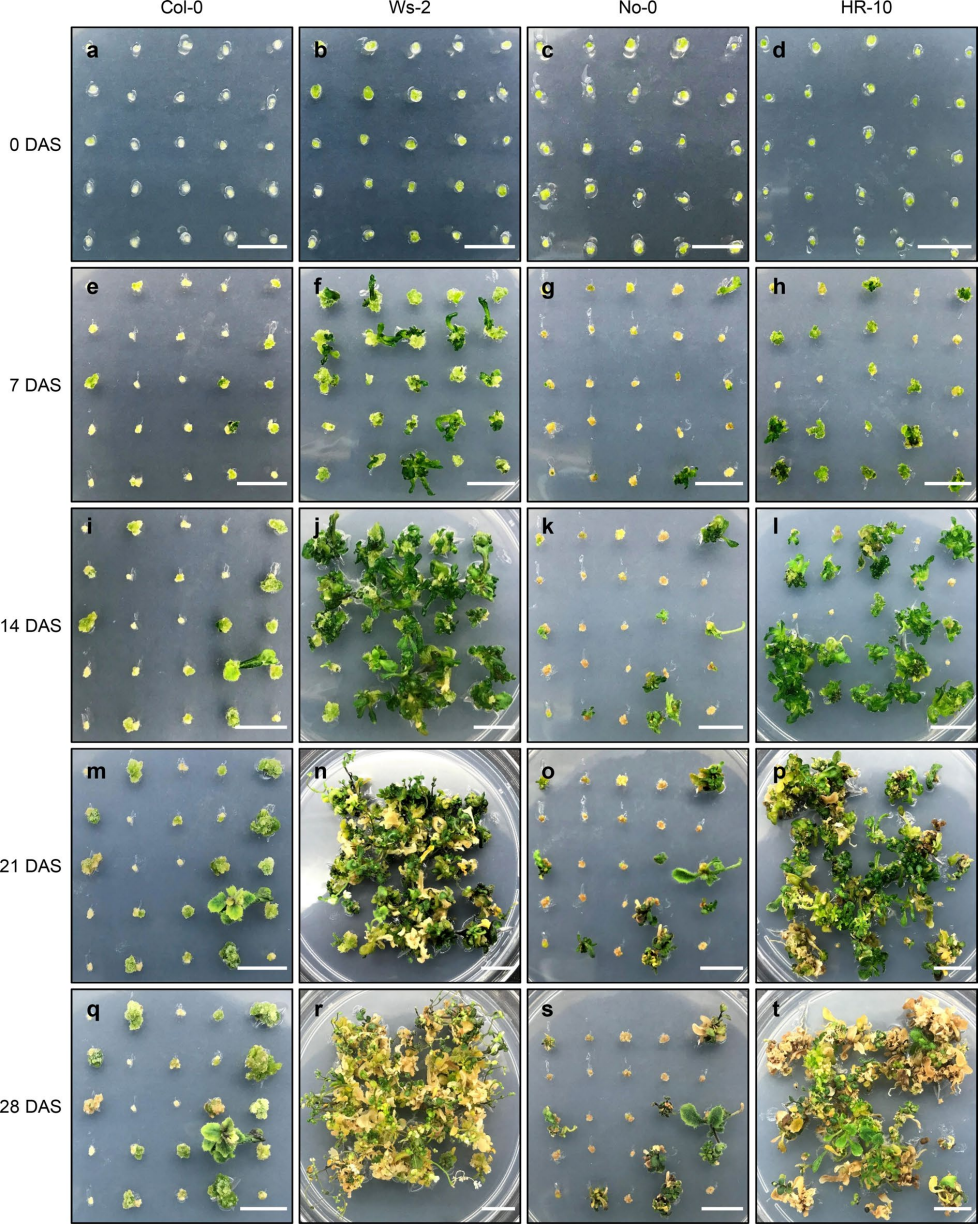
Shoot regeneration on SIM. **a**–**t** Images showing de novo shoot organogenesis at the indicated time points (days) after incubation on SIM (DAS): 0 DAS (**a**–**d**), 7 DAS (**e**–**h**), 14 DAS (**i**–**l**), 21 DAS (**m**–**p**), and 28 DAS (**q**–**t**). Scale bars = 1 cm

CIM-grown microcalli with a diameter of 1–2 mm were subjected to de novo shoot regeneration, although a significant number of shoots were already produced in the CIM (Table 4). Transfer to the solid medium for de novo shoot organogenesis facilitated shoot regeneration. However, *Arabidopsis* ecotypes displayed distinctive shoot regeneration efficiency: shoot regeneration was drastically increased in Ws-2 and HR-10, whereas Col-0 and No-0 continued to show low regeneration efficiency on SIM (Fig. 4a–t). Among all four ecotypes, Ws-2 showed the highest regeneration efficiency, reaching 100% shoot regeneration within 3 weeks on SIM, even when CIM-preincubated callus without shoot formation was used (Table 5).

**Table 5:** Shoot regeneration efficiency on three media for de novo shoot organogenesis

Ecotypes	Media		
	SIM (%)	SRMA (%)	SRA (%)
Col-0	18.67 ± 1.09	13.33 ± 2.88	6.67 ± 2.18
Ws-2	96.00 ± 3.27	93.33 ± 3.93	94.67 ± 2.88
No-0	30.67 ± 5.76	14.67 ± 6.62	10.67 ± 1.09
HR-10	76.00 ± 4.99	50.67 ± 3.93	12.00 ± 1.89

CIM-grown microcalli with a diameter of 1–2 mm were transferred to three different media for shoot regeneration. After 3-week incubation, shoot regeneration rate was calculated as the percentage of calli with emerging shoots relative to the total number of calli with a size of > 1 mm in a diameter. Data represent the mean ± SEM of biological triplicates

We also examined the shoot regeneration efficiency on three different media, including SIM [29], SRMA [17], and SRA [23]. All *Arabidopsis* ecotypes displayed the highest regeneration efficiency on SIM (Table 5). Counting of the number of regenerated leaves also revealed that SIM was the best medium for de novo shoot regeneration of calli derived from Ws-2 protoplasts (Table 6). Although shoot regeneration could be slightly improved by changing the medium, the genotype was a critical factor affecting the efficiency of de novo shoot organogenesis. Among four *Arabidopsis* ecotypes, Ws-2 ecotype exhibited the highest shoot regeneration rate on all media examined (Table 5).

**Table 6:** The average number of regenerated shoots from callus derived from Ws-2 protoplasts

	SIM	SRMA	SRA
Number of regenerated shoots/callus	15.63 ± 0.81	9.63 ± 0.27	8.39 ± 0.33

CIM-grown microcalli with a diameter of 1–2 mm were transferred to three different media for shoot regeneration. After 3-week incubation, the number of regenerated shoots from each callus was measured. Data represent mean ± SEM of biological triplicates

#### Protocol for de novo shoot regeneration from callus

1. Prepare 90 mm Petri dishes containing SIM supplemented with 0.8% agar.
2. Individually transfer each microcallus (diameter > 1 mm) onto the SIM using forceps.
3. Seal the SIM plate with 3 M tape and incubate the plates at 25 °C under continuous dim light conditions (50 µmol photons m^−2^ s^−1^) for 3 weeks.
  Note: Prolonged incubation on SIM results in leaf senescence and yellowing, which have a negative impact on de novo root organogenesis (Fig. 4r, t).

### Plantlet formation and reproduction

Following shoot regeneration, the shooting callus was subjected to de novo root organogenesis. Among four *Arabidopsis* ecotypes, only Ws-2 was used for subsequent analysis because the remaining three ecotypes (Col-0, No-0, and HR-10) showed low shoot regeneration efficiency (Table 5). We wanted to determine which part of the shooting callus should be used for de novo root regeneration. Because of precocious flowering [30], Ws-2 produced the inflorescence stem on the SIM. Thus, the inflorescence stem, regenerated vegetative leaf, and shooting callus of Ws-2 were excised and incubated on the RM, and the root regeneration rates were compared among the different explants (Additional file 5). Notably, the inflorescence stem explants showed the highest rooting rate, while leaf explants showed moderate rooting efficiency (Table 7). The shooting callus was significantly impaired in de novo root regeneration (Table 7).

**Table 7:** Rooting efficiency of various explants

Ecotypes	Media		
	MS (%)	RM (%)	RRA (%)
Inflorescence stem	60.34 ± 3.50	83.33 ± 2.62	N.D
Vegetative leaf	17.57 ± 1.79	34.89 ± 2.42	N.D
Shooting callus	6.83 ± 1.09	8.33 ± 1.96	N.D

Regenerated inflorescence stems, leaves, and shooting calli, were excised and transferred to three different media for root regeneration. After 3-week incubation, the percentage of root-regenerated explants relative to the total number of explants was calculated. Data represent the mean ± SEM of biological triplicates

*N.D.* not determined

**Fig. 5:**
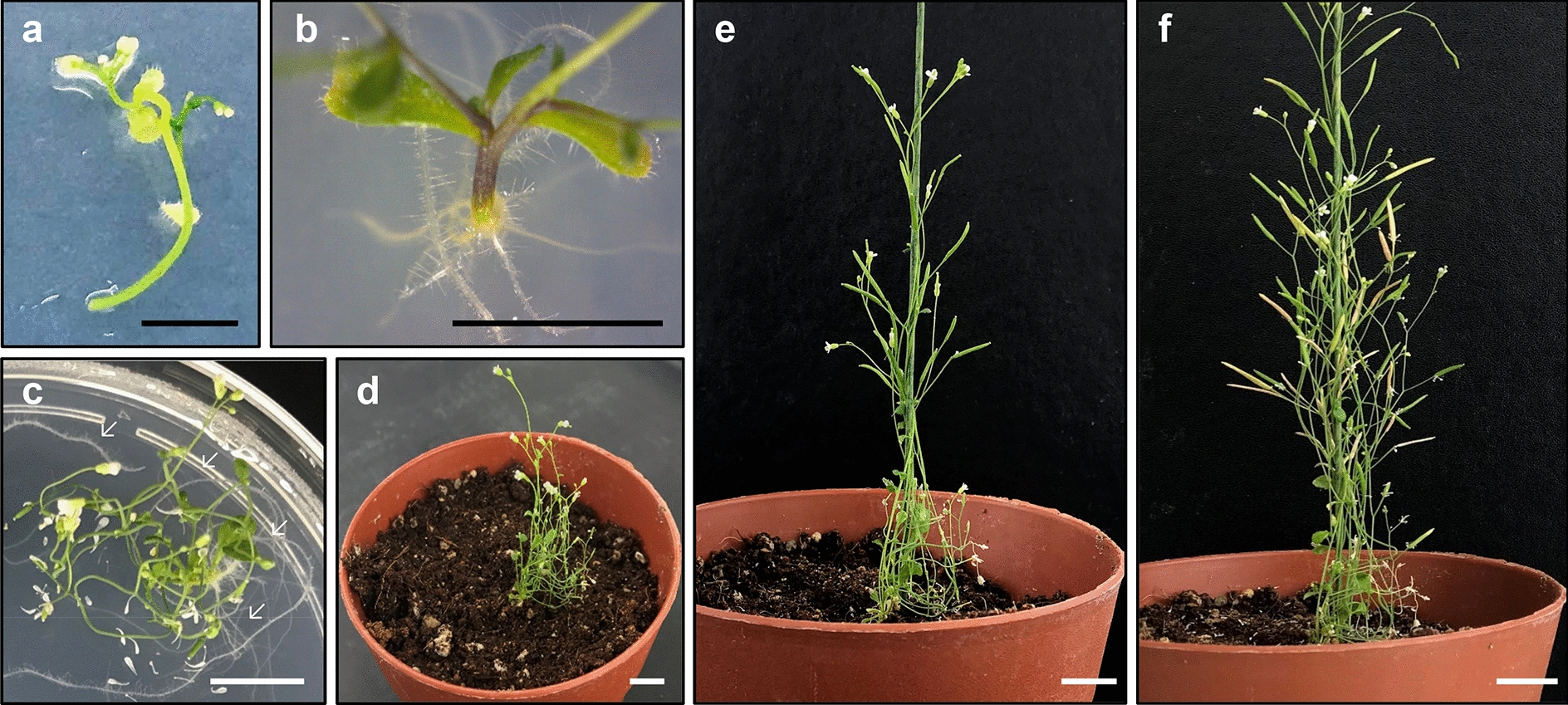
De novo root regeneration from inflorescence stem explants on RM. **a**–**c** Images showing de novo root organogenesis at the indicated time points (days) after incubation on RM (DAR): 0 DAR (**a)**, 12 DAR (**b**), and 21 DAR (**c**). In **c** arrows indicate regenerated roots. **d**–**f** Images of regenerated plantlets at the indicated time points (days) after soil-transfer (DAST): 7 DAST (**d**), 14 DAST (**e**), and 28 DAST (**f**). Black scale bars = 5 mm; white scale bars = 1 cm

We also tested the rooting efficiency on three different media for de novo root organogenesis (Table 7), MS, RM [17], and RRA [21]. Rooting efficiency was highest on RM (Table 7). While RRA also produced roots, regenerated roots by incubation on RRA were abnormal with dense root hairs (Additional file 6), which failed to grow in soil. Thus, we concluded that RM was the best medium for de novo root regeneration. Overall, the inflorescence stem of Ws-2 showed root regeneration within 2 weeks of incubation on RM (Fig. 5a–c).

Next, we transferred the regenerated plantlets to soil, and examined their growth and reproductive development. One-month after soil-transfer, the regenerated plantlets (R_0_) produced normal progeny (R_1_) (Fig. 5d–f). R_1_ seeds were sown in soil, and the growth and development of R_1_ plants were monitored. Post-embryonic growth of R_1_ plants was normal (Table 8, Additional file 7), demonstrating that protoplast regeneration produces fertile plants and morphologically normal progeny.

**Table 8:** Phenotypic analysis of progenies of plants regenerated from Ws-2 protoplasts

	Wild-type	R_1_ #1	R_1_ #2	R_1_ #3
Seed germination (%)	98.04 ± 0.80	97.06 ± 1.39	100	99.02 ± 0.80
Cotyledon area (mm^2^)	4.84 ± 0.15	4.88 ± 0.22	5.16 ± 0.22	4.93 ± 0.21
Flowering time (Rosette leaf no.)	8.37 ± 0.75	8.62 ± 0.85	8.27 ± 0.70	8.69 ± 0.67

Harvested R_1_ seeds from protoplast-regenerated plants were germinated on MS medium (> 60 seeds). Germination rate was calculated as the percentage of radicle emergence from the seed coat at 2 days after transfer to the long-day condition. Cotyledon area of 7-day-old seedlings was measured with ImageJ software. Flowering time was assessed as the number of rosette leaves, when the bolt reached 1 cm. Data represent the mean ± SEM of biological triplicates

#### De novo root regeneration protocol

1. Prepare 90 mm Petri dishes containing RM supplemented with 0.8% agar.
2. Excise inflorescence stems from the shooting callus and place the explants on RM.
  Note 1: The length of the inflorescence stem explant would be > 2 cm for de novo root organogenesis. Note 2: Callus tissue should be completely removed from explant, as it inhibits de novo root regeneration.
3. Seal the RM plate with 3 M tape, and incubate the plate at 23 °C under LD conditions for 3 weeks.
4. Transfer the regenerated plantlets (R_0_) to soil-filled pots.
  Note: Plantlets should be transferred to soil only when the root length has exceeded 5 cm.
5. Cover the pots with a plastic wrap for 5 days.
6. Acclimate the plantlets before removing the plastic wrap.
  Note: Because regenerated plantlets are extremely sensitive to rapid environmental changes, the acclimation step is necessary.
7. Harvest R_1_ seeds.

## Discussion

**Fig. 6:**
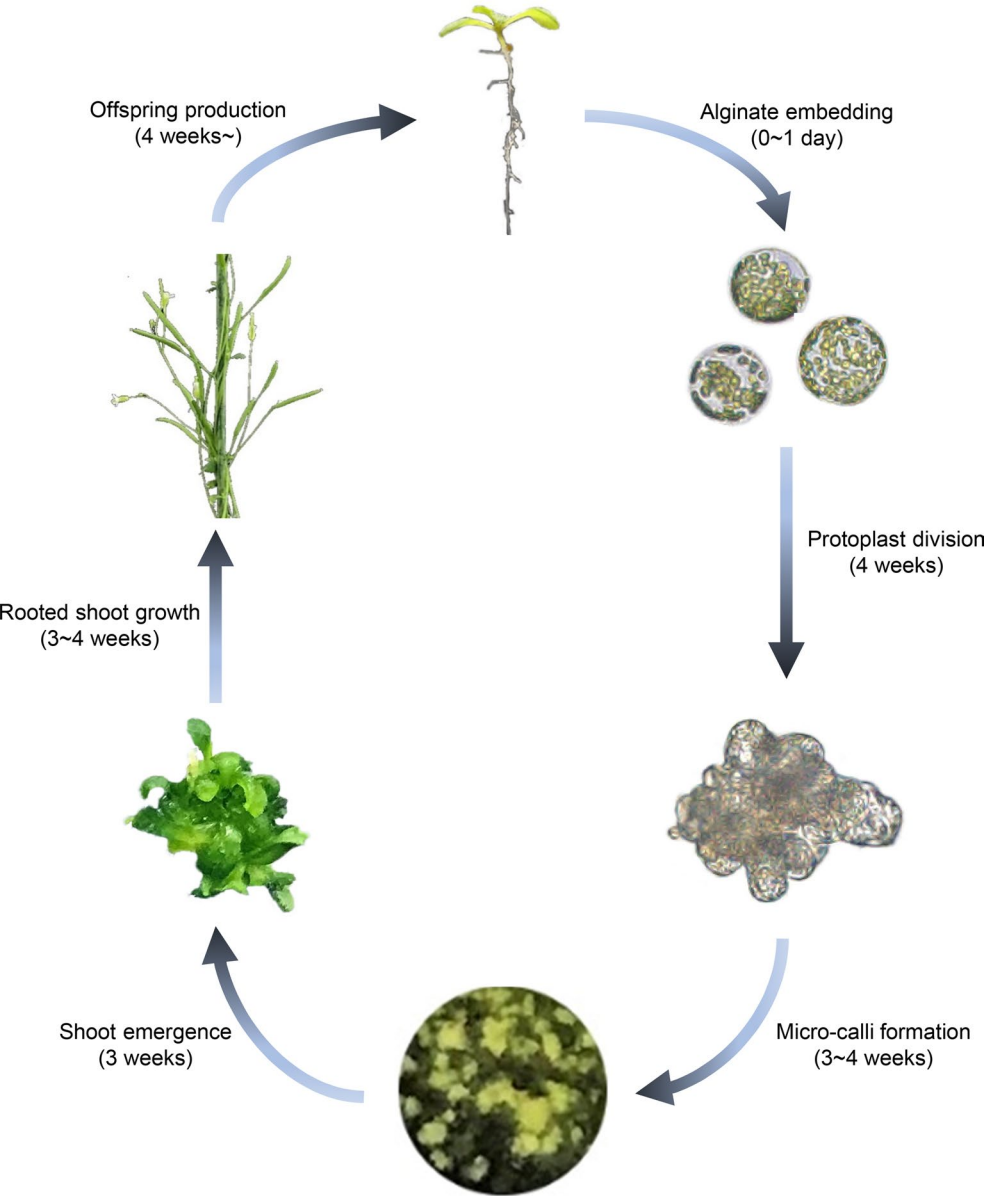
Schematic overview of the *Arabidopsis* protoplast regeneration process. Time required for each step is indicated in parentheses. Freshly isolated protoplasts are immobilized in a thin alginate hydrogel. Protoplasts start dividing upon incubation in PIM and produce callus by incubation in CIM. De novo shoot regeneration is facilitated by incubation on SIM for 3 weeks. Inflorescence stem explants are used for efficient de novo root regeneration. Plantlets are first grown on RM for 3 weeks to induce root regeneration and then transferred to soil to complete their life cycle and produce progenies. The whole process can be completed within 15 weeks

*Arabidopsis* protoplast regeneration has been previously demonstrated in a few studies [17, 21–24]. However, protoplast regeneration involves a series of intricate biological processes, and consistently, protoplast regeneration methods are usually labor-intensive. Thus, there are growing needs to make a reliable and simpler method. Here, we optimized the protocol for *Arabidopsis* protoplast regeneration with high efficiency and reproducibility (Fig. 6). Our protocol is composed of five main steps, including protoplast isolation, protoplast cell proliferation, microcallus formation, de novo shoot regeneration, and de novo root regeneration. Each step was optimized by examining multiple culture conditions. In particular, de novo root organogenesis has been a major hurdle in the protoplast regeneration process. Although it can be sometimes skipped, securing the large amount of progeny seeds requires de novo root organogenesis. We showed for the first time that incubation of inflorescence stem explants on RM, rather than that of callus or regenerated leaf explants, efficiently produced roots, leading to the regeneration of fertile plantlet. The regenerated plant progenies were morphologically normal, although we cannot exclude the possibility that the genome structure and epigenome landscape may have been altered during in vitro protoplast regeneration [31, 32].

We found that while technical advances could enhance the efficiency of protoplast regeneration, the protoplast regeneration rate was mainly determined by the genotype. *Arabidopsis* ecotype Ws-2 exhibited the most efficient protoplast regeneration, whereas the other ecotypes (Col-0, No-0, and HR-10) showed limited regeneration efficiency, which could not be overcome by changes in composition of media and tissue culture methods. These observations suggest that the genetic background must be screened to guarantee the success of plant regeneration in *Arabidopsis* as well as in other plant species.

It is noteworthy that protoplast regeneration is distinct from tissue explant-derived plant regeneration. Tissue explant-derived calli of HR-10 and No-0 are known to exhibit high shoot regeneration capabilities (Additional file 3) [25]; however, protoplast-derived microcalli of the two ecotypes exhibited relatively low regeneration efficiency. On the other hand, Ws-2, which shows moderate shoot regeneration efficiency from tissue explant-derived calli (Additional file 3) [26], displayed the highest regeneration efficiency during protoplast regeneration. These results indicate that protoplast regeneration and tissue explant-derived plant regeneration require different molecular processes, which is consistent with previous studies [10, 11].

Overall, we demonstrated an efficient protoplast regeneration protocol, which can be completed within 15 weeks. Given that other protocols usually take 6 months [17], our method can be used for various purposes including basic research related to cell wall biogenesis, cell proliferation, and de novo shoot and root regeneration. In addition, our protocol could also be used for comparing molecular processes underlying protoplast regeneration and tissue explant-derived plant regeneration. A comprehensive understanding of protoplast regeneration will create further opportunities for protoplast-based biotechnology applications such as CRISPR-based genome engineering.

## Conclusions

An efficient and reproducible *Arabidopsis* protoplast regeneration protocol was developed in this study. This protocol comprised five main steps, and various in vitro culture conditions were examined for the optimization of each step. Efficient protoplast regeneration of a model plant *Arabidopsis* will allow fundamental studies related to cell–cell interactions, cell wall biogenesis, cell cycle re-entry, pluripotency acquisition of microcallus, and de novo tissue organogenesis.

## Supplementary Information

**Additional file 1.** Chemicals used in study.

**Additional file 2.** Equipment used in study.

**Additional file 3.** Plant regeneration using hypocotyl explants.

**Additional file 4.** Effect of protoplast density in alginate hydrogels on protoplast division.

**Additional file 5.** De novo root regeneration efficiency of different tissues.

**Additional file 6.** De novo root regeneration of inflorescence explants on three different root induction media.

**Additional file 7.** Phenotypic comparison of wild-type and progeny (R_1_) of protoplast-regenerated plants.

## Data Availability

All data generated or analyzed during this study are included in this published article and its Additional files.

## Abbreviations

CRISPR: Clustered regularly interspaced short palindromic repeat
Cas9: CRISPR-associated protein 9
RNP: Ribonucleoprotein
PEG: Polyethylene glycol
Col: Columbia
Ws: Wassilewskija
No: Nossen
MMC: MES, Mannitol, Calcium
DAP: Days after incubation in PIM
PIM: Protoplast Induction Medium
CMA: Culture Medium A
PCA: Protoplast culture *Arabidopsis*
DAC: Days after incubation in CIM
CIM: Callus Induction Medium
CMC: Culture Medium C
DAS: Days after incubation on SIM
SIM: Shoot Induction Medium
SRMA: Shoot Regeneration Medium A
SRA: Shoot Regeneration *Arabidopsis*
DAR: Days after incubation on RM
RM: Rooting Medium
RRA: Root Regeneration *Arabidopsis*
DAST: Days after soil-transfer
