# Supplementary material for: Optimization of protoplast regeneration in the model plant Arabidopsis thaliana
Source: Plant Methods. 2021 Feb 23;17:21. doi: 10.1186/s13007-021-00720-x (PMC7901198; doi:10.1186/s13007-021-00720-x)
Supplement: Supplementary file 1 — Additional file 1. Chemicals used in study. [file 13007_2021_720_MOESM1_ESM.pdf]

Additional file 1

Reagents used in study

- Viscozyme L (Novozymes, cat. no. SP079)
- Pectinex Ultra SP-L (Novozymes, cat. no. 1043-70)
- Celluclast 1.5L (Novozymes, cat. no. SP011)
- MES monohydrate (Duchefa Biochemie, cat. no. M1503)
- $CaCl_2$ (Junsei, cat. no. 18235-0301)
- $MgSO_4 \cdot 7H_2O$ (Merck, cat. no. 1.05886)
- L(+)-Glutamine (Junsei, cat. no. 27340-0310)
- D-mannitol (Junsei, cat. no. 70220-0301)
- NaOH (Sigma-Aldrich, cat. no. S8045)
- Sucrose (Sigma-Aldrich, cat. no. S1888)
- D-glucose (Sigma-Aldrich, cat. no. G7021)
- Coconut water (Sigma-Aldrich, cat. no. C5915)
- Sodium alginate (Sigma-Aldrich, cat. no. A0682)
- Plant agar (Duchefa Biochemie, cat. no. P1001)
- Gamborg B5 medium including vitamins (Duchefa Biochemie, Cat. no. G0210)
- Murashige & Skoog medium including vitamins (Duchefa Biochemie, Cat. no. M0222)
- Myo-inositol (Duchefa Biochemie, cat. no. I0609)
- 6-Benzylaminopurine (6-BAP) (Duchefa Biochemie, cat. no. B0904)
- α -Naphtalene acetic acid (α -NAA) (Duchefa Biochemie, cat. no. N0903)
- Indole-3-butyric acid (IBA) (Duchefa Biochemie, cat. no. I0902)
- Indole-3-acetic acid (IAA) (Duchefa Biochemie, cat. no. I0901)
- 6-(γ,γ-Dimethylallylamino)purine (2-ip) (Sigma-Aldrich, cat. no. D7674)
- 2,4-Dichlorophenoxyacetic acid (2,4-D) (Duchefa Biochemie, cat. no. D0911)
- Ethanol (Merck, cat. no. 8.18760)
- Triton X-100 (Sigma-Aldrich, cat. no. T9284)

Additional file 1. Chemicals used in study
