# Supplementary material for: Optimization of protoplast regeneration in the model plant Arabidopsis thaliana
Source: Plant Methods. 2021 Feb 23;17:21. doi: 10.1186/s13007-021-00720-x (PMC7901198; doi:10.1186/s13007-021-00720-x)
Supplement: Supplementary file 2 — Additional file 2. Equipment used in study. [file 13007_2021_720_MOESM2_ESM.pdf]

Additional file 2

Equipment used in study

- Pipette-aid (HDR-4-000-201; Drummond)
- 10 ml serological pipette (SPL, cat. no. 91010)
- 30 ml syringe (Kovax)
- 0.2 μm syringe sterilization filter (Sartorius, cat. no. 16534K)
- 40 μm cell strainer (SPL, cat. no. 93040)
- 100 mm plate (SPL, cat. no. 10100)
- 90 mm plate (SPL, cat. no. 20100)
- 60 mm plate (SPL, cat. no. 20060)
- 3M micropore tape (3M, cat. no. 1530-0)
- Parafilm (Sigma-Aldrich, cat. no. PM996)
- 14 ml round-bottomed tube (SPL, cat. no. 40014)
- Hemocytometer (Marienfeld)
- Shaker (VS-201D; Vision scientific)
- Double room incubator (HB-201MS-2R; Hanbaek scientific technology)
- LED incubator (VS-1203P5-L0; Vision scientific)
- Optical microscope (IX53; Olympus)
- Table-top centrifuge (Combi 514R; Hanil)
- Autoclave (VS-1221; Vision scientific)
- Growth incubator (VS-1203P5-LO; Vision scientific)
- Growth incubator (VS-3125Di; Vision scientific)
- Clean bench (VS-7120LH; Vision scientific)
- Stainless-steel spatula (Scilab, cat. no. SL.sap7012)
- Stainless-steel forceps (Scilab, cat. no. SL.For7076)
- Stainless-steel Scalpel blade (Feather, cat. no. HFE-SB10)
- Stainless-steel Scalpel handle (Feather, cat. no. 72040-03)

Additional file 2. Equipment used in study
