# Supplementary material for: Optimization of protoplast regeneration in the model plant Arabidopsis thaliana
Source: Plant Methods. 2021 Feb 23;17:21. doi: 10.1186/s13007-021-00720-x (PMC7901198; doi:10.1186/s13007-021-00720-x)
Supplement: Supplementary file 3 — Additional file 3. Plant regeneration using hypocotyl explants. [file 13007_2021_720_MOESM3_ESM.pdf]

Additional file 3

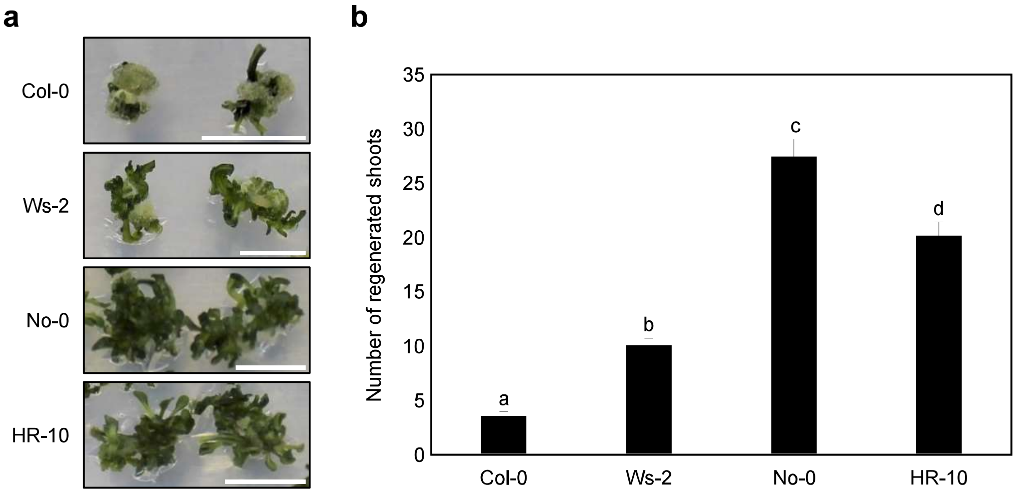

Additional file 3. Plant regeneration using hypocotyl explants.
Calli preincubated for 7 days on callus-inducing medium (MS salt, 3% sucrose, 0.05% MES.H_2O, 0.5 mg/L 2,4-D, 0.05 mg/L kinetin, pH 5.8, and 0.8% plant agar) in darkness were used to induce shoot regeneration on shoot-inducing medium (MS salt, 3% sucrose, 0.05% MES.H_2O, 0.1576 mg/L IAA, 0.501 mg/L 2-IP, pH 5.8, and 0.8% plant agar) ($n \geq 30$). Plates were incubated for 2 weeks under continuous light conditions and photographed (**a**). Scale bars = 1 cm. The number of regenerated shoots from each callus was measured ($n \geq 30$) at 2 weeks after incubation on shoot-inducing medium (**b**). Biological triplicates were averaged. Different letters represent a significant difference at $P < 0.05$ (one-way ANOVA with Fisher's *post hoc* test). Bars indicate the standard error of the mean.
