# Supplementary material for: Optimization of protoplast regeneration in the model plant Arabidopsis thaliana
Source: Plant Methods. 2021 Feb 23;17:21. doi: 10.1186/s13007-021-00720-x (PMC7901198; doi:10.1186/s13007-021-00720-x)
Supplement: Supplementary file 4 — Additional file 4. Effect of protoplast density in alginate hydrogels on protoplast division. [file 13007_2021_720_MOESM4_ESM.pdf]

Additional file 4

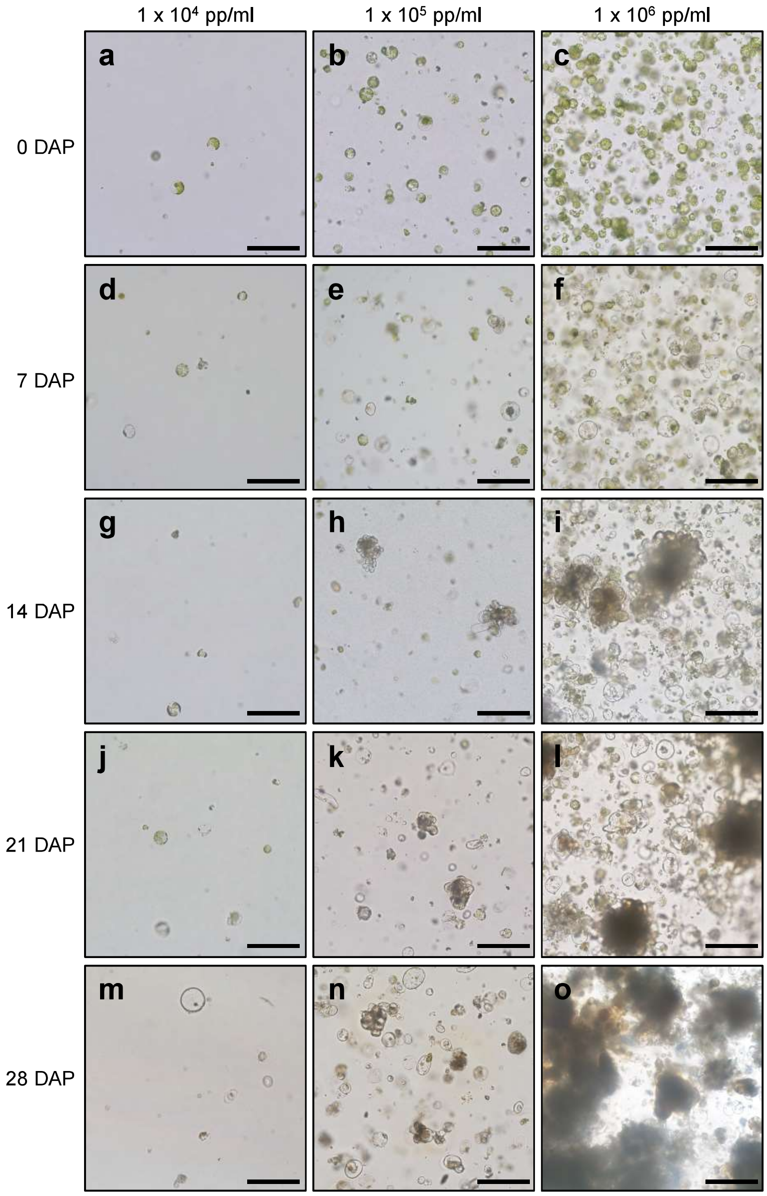

Additional file 4. Effect of protoplast density in alginate hydrogels on protoplast division. Protoplast images were taken at the indicated time points (days) after incubation in PIM (DAP): 0 DAP (**a–c**), 7 DAP (**d–f**), 14 DAP (**g–i**), 21 DAP (**j–l**), and 28 DAP (**m–o**). Scale bars = 200 mm. DAP, days after incubation in protoplast induction medium (PIM); pp, protoplasts.
