# Supplementary material for: Optimization of protoplast regeneration in the model plant Arabidopsis thaliana
Source: Plant Methods. 2021 Feb 23;17:21. doi: 10.1186/s13007-021-00720-x (PMC7901198; doi:10.1186/s13007-021-00720-x)
Supplement: Supplementary file 5 — Additional file 5. De novo root regeneration efficiency of different tissues. [file 13007_2021_720_MOESM5_ESM.pdf]

Additional file 5

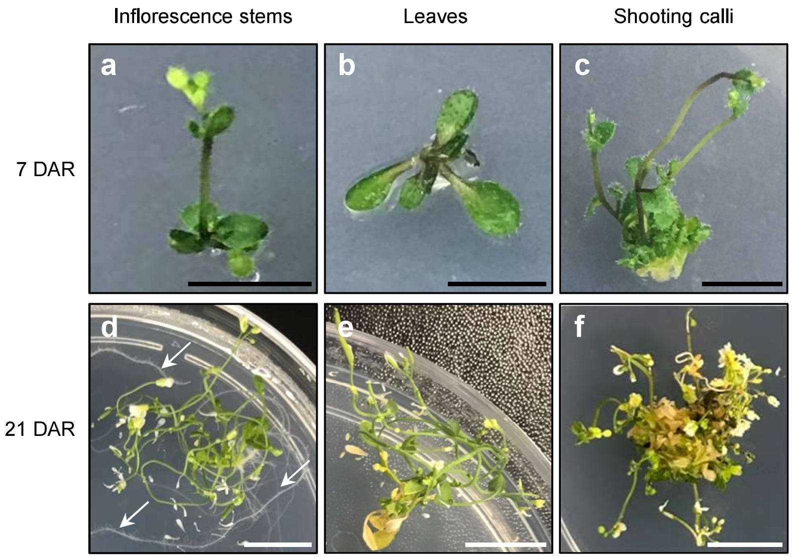

Additional file 5. *De novo* root regeneration efficiency of different tissues.
Regenerated inflorescence stems (**a**), vegetative leaves (**b**), and shooting calli (**c**) were excised and incubated on RM. Images of *de novo* root regeneration for each tissue (**d-f**) were taken at the 21 days after incubation on RM (DAR). Arrows indicate *de novo* regenerated roots. Black scale bars = 5 mm; White scale bars = 1 cm.
