# Supplementary material for: Optimization of protoplast regeneration in the model plant Arabidopsis thaliana
Source: Plant Methods. 2021 Feb 23;17:21. doi: 10.1186/s13007-021-00720-x (PMC7901198; doi:10.1186/s13007-021-00720-x)
Supplement: Supplementary file 6 — Additional file 6. De novo root regeneration of inflorescence explants on three different root induction media. [file 13007_2021_720_MOESM6_ESM.pdf]

Additional file 6

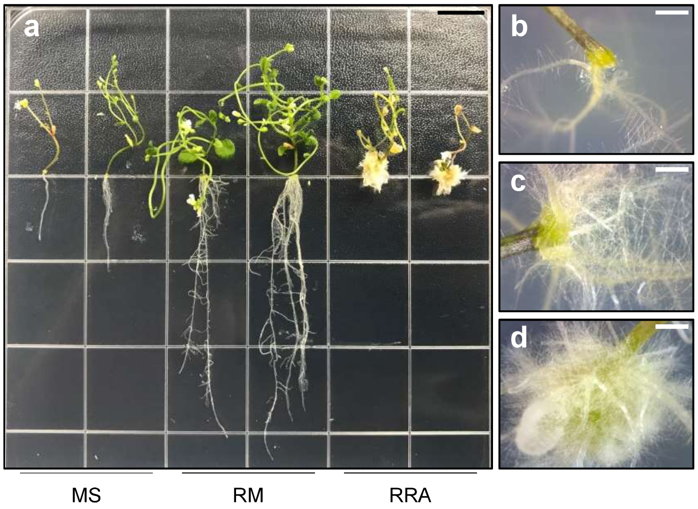

Additional file 6. *De novo* root regeneration of inflorescence explants on three different root induction media.
(**a**) Regenerated inflorescence stems of *Arabidopsis* ecotype Ws-2 on three different root induction media. The inflorescence stems of Ws-2 were excised and incubated on each indicated root induction medium for 2 weeks. Enlarged root images on MS (**b**), RM (**c**), and RRA (**d**) are shown. Black scale bars = 1 cm; white scale bars = 1 mm.
