# Supplementary material for: Optimization of protoplast regeneration in the model plant Arabidopsis thaliana
Source: Plant Methods. 2021 Feb 23;17:21. doi: 10.1186/s13007-021-00720-x (PMC7901198; doi:10.1186/s13007-021-00720-x)
Supplement: Supplementary file 7 — Additional file 7. Phenotypic comparison of wild-type and progeny (R1) of protoplast-regenerated plants. [file 13007_2021_720_MOESM7_ESM.pdf]

Additional file 7

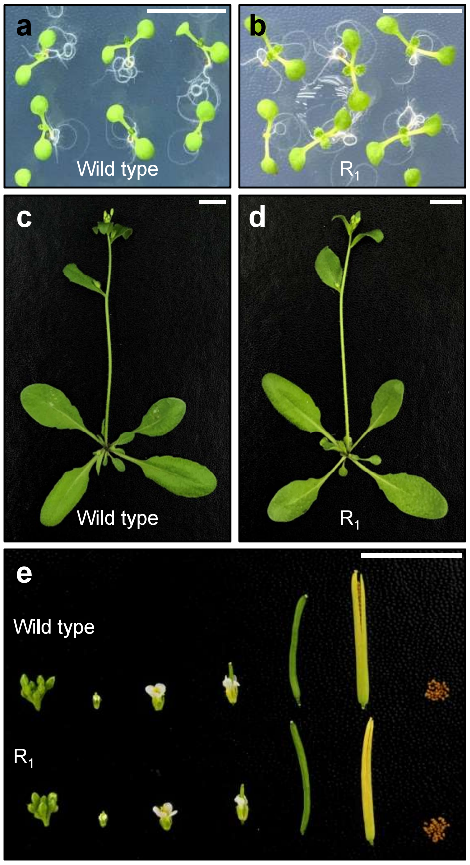

Additional file 7. Phenotypic comparison of wild-type and progeny (R_1) of protoplast-regenerated plants.

(**a, b**) Seed germination. Wild-type (**a**) and R_1 (**b**) seeds were germinated on MS medium, incubated for 7 days under long-day conditions, and photographed.

(**c, d**) Flowering phenotypes. Wild-type (**c**) and R_1 (**d**) seeds were sown in soil and grown for 3 weeks under long-day conditions.

(**e**) Flower and silique development. Plants were grown in soil and grown for 8 weeks under long-day conditions. Scale bars = 1 cm.
